# Criterion Validity of the Newly Developed Occlusal Cant Index

**DOI:** 10.3390/ijerph191811623

**Published:** 2022-09-15

**Authors:** Hessah A. Alhuwaish, Khalid A. Almoammar, Abdulaziz S. Fakhouri, Lamya M. Alabdulkarim

**Affiliations:** 1 Department of Pediatric Dentistry and Orthodontics, College of Dentistry, King Saud University, Riyadh 11451, Saudi Arabia; 2 Department of Biomedical Technology, College of Applied Medical Sciences, King Saud University, Riyadh 11451, Saudi Arabia; 3 Department of Health Rehabilitation Sciences, College of Applied Medical Sciences, King Saud University, Riyadh 11451, Saudi Arabia

**Keywords:** index, occlusal cant, criteria validation, index development

## Abstract

Objectives: To assess the criterion-related (concurrent) validity of the newly developed occlusal cant index (OCI). Materials and Methods: Four standardized posterior–anterior (PA) cephalometric radiographs of four patients were obtained at a 0° occlusal cant (OC) and manipulated to create various degrees of OC from 1° to 4° on the right and left sides, with a total of 36 PA images. The angle between the actual horizontal line and the occlusal plane was manually drawn on each PA radiographic image. The set of radiographic images was displayed to 36 orthodontists, who were asked to measure the drawn angle and apply the OCI to each PA radiographic image. Results: The overall criterion-related validity of the OCI was statistically significant among all grades. Conclusion: The OCI is highly valid and recommended for clinical consideration.

## 1. Introduction

Occlusal cant (OC) is a malocclusion trait known as the rotation of the occlusal plane (OP) in the transverse direction [1]. Little emphasis has been placed on OC in the literature compared to other anteroposterior, vertical, and transverse malocclusion characteristics. In the index of orthodontic treatment need, which is regarded as one of the commonest indices, the tilt in OP has not been considered in any of the categories [2]. Only recently, in an index concerned with orthognathic functional needs, OC was considered in category 2, which reflects little need, despite labeling OC as a marked discrepancy [3]. Peer assessment rating—a method of producing an index to assess the quality of treatment outcomes—was introduced in 1992 [4]. Arch crowding, anteroposterior molar relationship, overjet, and overbite were also evaluated and rated. In the transverse plane, midlines and crossbite were considered and weighted. However, there was no mention of OC in the pre-treatment or post-treatment scores [4]. These issues reflect the minimal emphasis on OC in orthodontic indices.

Recently, a new index for occlusal cant was developed to guide practitioners in communication, diagnosis, and research (Table 1) [5]. The categorization of the occlusal cant index (OCI) was based on OC detection among orthodontists and laypersons [5]. It consists of four grades, as follows:

- Grade 0 where 0 represents the absence of an OC, and the OP is parallel to the true horizontal plane;
- Grade I refers to a mild OC of 1°;
- Grade II involves an OC range from 2° to 3°;
- Grade III consists of severe OC cases of 4° and above (Table 1) [5].

According to the World Health Organization (WHO) (1977), an ideal healthcare index should be valid [6]. Validity is defined as “the degree of which the method measures what it is meant to measure” and has three main types in quantitative research [7], as follows:

- Content validity is an expert’s subjective judgment about the degree of representativeness, relevance, and clarity or completeness of each item of measurement or tool on a four-point scale [8,9,10,11]. This assessment is the primary step for complete validation of any newly developed index or scale;
- Criterion-related validity is the relationship between the use of a specific criterion and the new measurement method [7,12]. This type of validity shows the closest relationship between an experimental test and a reference criterion standard [7,13]. Criterion-related validity has two primary forms: (1) concurrent validity, the correlation or application of a newly developed instrument or tool, and an existing standard (gold standard), and (2) predictive validity, which refers to the degree to which an instrument can forecast an outcome;
- Construct validity is the ability of the instrument to measure the intended theoretical construct [7,14,15]. Construct validity is considered the most challenging measure of validity because it utilizes a hypothetical construct for comparison [12,15].

The OCI was validated using content validity as the primary step in the validation process and scored with high validity based on the content validity index (CVI), including both the item-level CVI (I-CVI) and the scale-level CVI (S-CVI). To complete the route of the newly developed OCI validation process to have a valid dental healthcare index, further objective validation assessment should be conducted. This study aimed to assess the criterion validity (concurrent) of the OCI. The null hypothesis is that the OCI is not valid when criterion validations are applied.

## 2. Methods

### 2.1. Radiograph Selection and Preparation

A retrospective study was conducted at the College of Dentistry at King Saud University. Ethical approval (No. E-21-5905) for the study protocol was obtained from the Institutional Review Board (IRB) of King Saud University Dental Hospital. The study was approved and registered at the College of Dentistry Research Center, College of Dentistry, King Saud University (No. 0123). Posterior–anterior (PA) cephalometric radiographs were collected from the Department of Oral and Maxillofacial Surgery database between December 2021 and February 2022. A total of 22 PA cephalometric radiographs were identified, and their allocation was based on the following criteria:

1. Adults aged 18 years or older;
2. Absence of any facial asymmetry features, including OC;
3. Exposure to the same equipment (Planmeca ProMax^®^, Helsinki, Finland) with a standardized technique (patients in natural head position and teeth in centric occlusion);
4. No craniofacial syndromes;
5. No history of maxillofacial surgery;
6. Absence of intra-oral metallic objects, such as a dental implant, orthodontic brackets, and amalgam restorations;
7. No history of tooth extraction.

**Figure 1:**
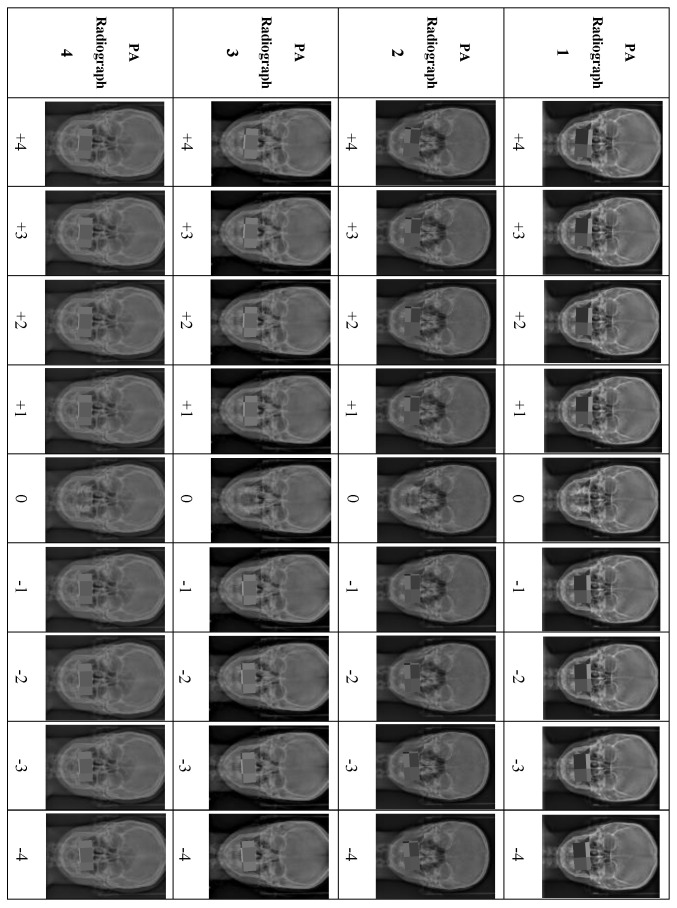
Illustration of the manipulations of all the PA cephalometric radiographs, +1° to +5° (OC at the left side), and −1° to −5° (OC at the right side).

Eighteen PA cephalometric radiographs were excluded because they did not meet the inclusion criteria. Four PA cephalometric radiographs, representing two men and two women, were included in the study. Written informed consent was obtained from the four patients to use their PA radiographs with all the desired manipulations. For each PA radiograph, the upper and lower teeth in occlusion were cropped and manipulated to create different degrees of OC using Photoshop (Adobe Photoshop 9.0, San Jose, CA, USA). For accurate manipulation, the true horizontal line was used as a reference to rotate the OP in each PA image digitally. The OP in the original PA image was rotated in 1° increments from 1° to 4° in both directions. Accordingly, a set of nine manipulated PA images exhibiting various degrees of OCs based on the cut-off points of the newly developed OCI were prepared from each PA image. Each PA radiograph was manipulated to produce eight PA images representing Grades I to III of the OCI, with 1° representing Grade I, 2° and 3° representing Grade II, and 4° of OC representing Grade III (four right-sided and four left-sided), and the ninth manipulated PA image representing Grade 0 (0°). Thus, the four PA radiographs produced 36 manipulated PA images (Figure 1).

**Figure 2:**
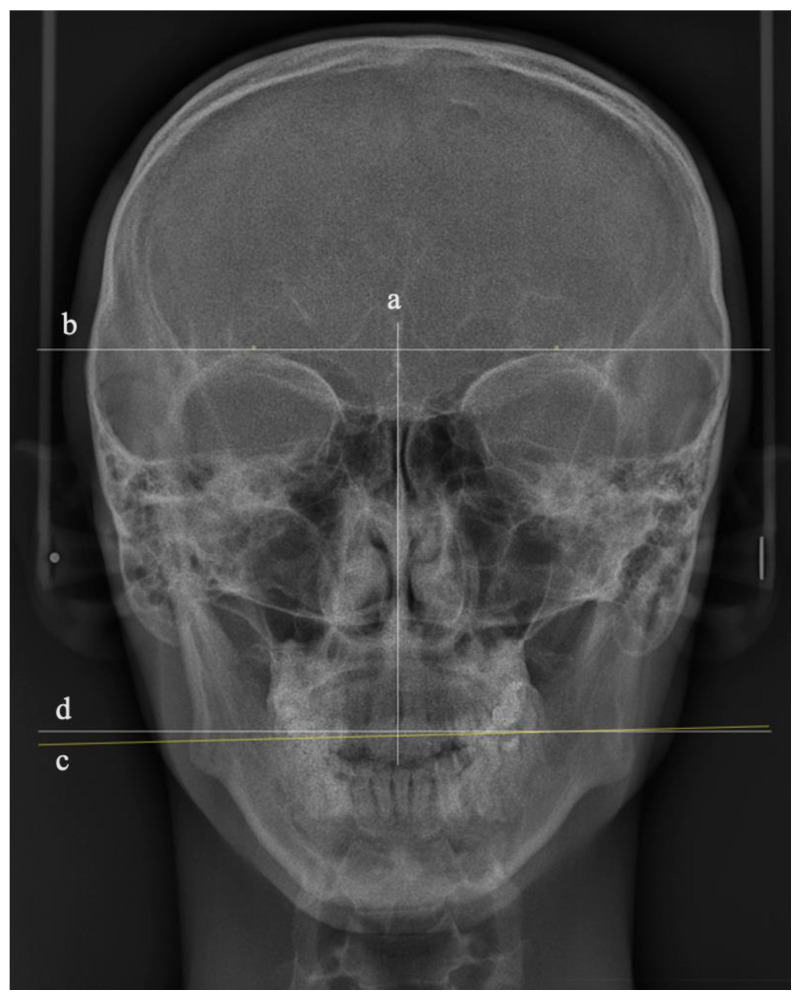
Illustration of the tracing lines of the PA cephalometric images: (**a**) vertical line representing the facial midline, (**b**) true horizontal line, (**c**) occlusal plane line, and (**d**) tangent parallel to the true horizontal line.

The manipulated 36 PA images were printed and traced by a single author. To measure the OC cephalometrically, three lines were drawn using a fine-tip pen (0.20 mm) (Sakura Pigman micron pen, Osaka, Japan), as shown in Figure 2:

1. A vertical line represents the facial midline, drawn from the crista galli to a point in the upper third of the nasal septum;
2. A line represents the true horizontal plane, drawn as a tangent passing through the supra-orbital rims perpendicular to the vertical line;
3. A line represents the occlusal plane (OP), drawn as a tangent to the most convex point of the buccal surfaces of the upper right and left first molars.

The degree of OC was represented by the angle bisecting these two horizontal lines [16]. Considering the distance between the two horizontal lines, an additional line parallel to the true horizontal tangent was drawn closer to the OP tangent to measure the OC angle (Figure 2). To assess the accuracy of the tracing, the angle of OC in degrees for all the traced 36 PA cephalometric images was remeasured by a second researcher. The set of 36 PA cephalometric images was arranged randomly to reduce systematic measurement errors among the participants, enhancing the study’s internal validity.

### 2.2. Panel of Experts and Data Collection

A panel of 36 orthodontists with at least three years of clinical experience was randomly selected from the dental college of King Saud University. Informed consent was obtained from each orthodontist to participate in the study. The validation process was initiated by presenting the newly developed OCI table, which was explained to the experts in a short five-minute presentation. Next, 36 prepared and randomly arranged PA cephalometric images were presented. Each participant was directed to manually measure the traced OC angle and then classify the OC at each PA cephalometric image using the newly developed OCI. To measure the criterion validity of the newly developed OCI, the orthodontists’ performances on the OCI were compared at the same time interval to the actual OC angles and their ideal classification. For inter- and intra-examiner reliability assessment, 10 participants were randomly selected to remeasure and reclassify all PA cephalometric radiograph images at two-week intervals.

## 3. Statistical Analysis

All data were entered and analyzed using the Statistical Package for the Social Sciences (SPSS) software, version 26.0 (IBM Inc., Chicago, IL, USA). Descriptive statistics (means, standard deviations, and frequencies) were used to describe all quantitative variables. The significant difference in the criterion validity assessment of OCI at (*p* = 0.05) was calculated using the chi-squared test. Kappa statistics were used to assess the inter-researcher reliability in measuring the traced angles in each PA radiograph image. This test was used again to assess the inter- and intra-examiner reliabilities in the OCI validity processes.

## 4. Results

A sample size estimation based on a power of 0.9 at a *p*-value of 0.05 confirmed that the required number of participants to be enrolled was 36. The inter-researcher reliability of measuring the traced angles showed a high kappa value of 0.85. Furthermore, the inter- and intra-examiner reliability among the orthodontists in the index validity assessments showed high kappa values of 0.84 and 0.80, respectively.

The overall responses of the participants showed the statistically significant validity of the newly developed OCI. Table 2 shows 100% correct responses among participants, indicating a statistically significant criterion validity in Grade 0 (0°) of OCI. Identifying Grade I (1°) in OCI was successful in both diagnosis and direction, with a 3% lower ability to identify the left side compared to the right side (Table 3). The overall validity of Grade II in OCI (which includes 2° and 3° of OC) was statistically significant among all orthodontists, with no difference between the right and left sides (Table 4). Table 5 shows very high validity in identifying Grade III in both diagnosis and direction.

## 5. Discussion

OC is an unpleasant feature of a smile that influences the tooth show and the smile arc [17,18]. In severe forms, this transverse discrepancy requires surgical intervention [19,20]. Previous literature revealed a limitation in OC categorization among clinical orthodontics assessment indices. The OCI was recently developed to categorize OC to facilitate communication and diagnosis. Furthermore, OCI can be utilized for research purposes [5]. Following the development of the OCI, content validation by expert opinion was conducted as an initial step towards a complete validation of the instrument [5]. Content validation of the current index was used to confirm the instrument’s suitability as a simple diagnostic tool in communication, diagnosis, and future modifications. Hence, this study aimed to measure the criterion validity of the newly developed OCI by a panel of experts using criterion validation. The null hypothesis that OCI is invalid when applying the criterion for validation is rejected.

Criterion validation is a form of testing a new tool or instrument as the gold standard [7,12]. The responses of the 36 expert participants showed the highly significant validity of the newly developed index. All participants were anticipated to successfully diagnose and categorize Grade 0, indicating no tilt in the OP. The criterion validation process underlines both diagnostic assessment and direction. For this reason, most participants were correct in diagnosing Grade I. In two attempts, the participants made a faulty diagnosis of the cant (Grade I). Such a false diagnosis, while insignificant, may be attributed to the small degree of difference between no cant and one degree of OC, which corresponds to Grade I. In addition, three instances of faulty assessment occurred on the left side. A similar rate of direction-related mistakes was noticed in Grade II, with a total of five errors, and in Grade III, on two occasions. Minor errors in categorization were excluded when undertaking many measurements if substantial validation was achieved. These minor errors may be attributed to participants’ fatigue and level of concentration. The inter- and intra-examiner reliabilities among the participants showed a high kappa value, reflecting the clear consistency and reproducibility of the participants’ efforts.

A cant in the OC appears upon smiling. Still, it is not possible to detect using diagnostic records, such as study models and intra-oral photography, since the smile arc is not related to anatomical landmarks [21]. In this study, the participants used a posteroanterior radiograph to diagnose OC. The selected radiographs excluded any variables affecting the accuracy of landmark identifications and, consequently, tracing, and measurements. It was essential to select posteroanterior radiographs of patients with no metallic inserts, orthodontic brackets, surgical plates, or extensive restorations, which would have compromised the clarity of the structures.

The true horizontal line was used as a horizontal reference to assess the OP [16]. Furthermore, manipulations were prepared around this true horizontal line, representing the different categories of the proposed index. The inter-researcher reliability of measuring the traced angles in each manipulated PA cephalometric image showed high reliability, indicating the considerable accuracy of the traced angles. The panel of experts underwent a standardized participation and assessment procedure. All participants were given a five-minute presentation explaining the newly proposed index; a copy of the index was printed during the assessment procedure. The entire procedure was conducted in a quiet room with sufficient lighting. The panel consisted of orthodontists with a minimum of three years of experience. This level of standardization was intended to reduce bias in the validation process.

One limitation of this study was the lack of generalizability of the results because the validation process was performed in only one region. The authors recommend an international agreement using a multi-centered approach to gain global recognition. Further, future modifications to the index may involve the origin of OC and treatment possibilities.

## 6. Conclusions

This study confirmed that the newly developed index for diagnosing OC is highly valid. This high (criterion) validity of the diagnosis of occlusal cant is statistically confirmed by a group of experienced orthodontists. Accordingly, we believe this index should be considered a clinically usable methodology to aid in inter-professional communication, diagnosis, and dental professional training.

## Figures and Tables

**Table 1:** Occlusal Cant Index (OCI) [5].

Grades	Degree Side		Descriptions
Grade 0	0°		No OC is present (the OP is parallel to the true horizontal plane)
Grade I	1°	Right	The OP is tilted down on the right side by 1°
	1°	Left	The OP is tilted down on the left side by 1°
Grade II	2–3°	Right	The OP is tilted down on the right side by 2–3°
	2–3°	Left	The OP is tilted down on the left side by 2–3°
Grade III	≥4°	Right	The OP is tilted down on the right side by ≥4°
	≥4°	Left	The OP is tilted down on the left side by ≥4°

**Table 2:** Validity of OCI at Grade 0.

Answer	Count	%
Correct	144	100%
Error	0	0%
Total	144	100%

**Table 3:** Validity of OCI at Grade I (1°).

Answer	RIGHT				LEFT				ALL			
	Diagnosis	%	Direction	%	Diagnosis	%	Direction	%	Diagnosis	%	Direction	%
Correct	143	99	144	100	143	99	140	97	286	99	284	99
Error	1	1	0	0	1	1	4	3	2	1	4	1
Total	144	100	144	100	144	100	144	100	288	100	288	100
Chi-Square	140.028				140.028		128.44		280.056		272.22	
*p*-Value	0.00 ***				0.00 ***		0.00 ***		0.00 ***		0.00 ***	

*** = *p* ≤ 0.001.

**Table 4:** Validity of OCI at Grade II (2° and 3°).

Answer	RIGHT				LEFT				ALL			
	Diagnosis	%	Direction	%	Diagnosis	%	Direction	%	Diagnosis	%	Direction	%
Correct	288	100	285	98.96	288	100	286	99.31	576	100	571	99.48
Error	0	0	3	1.04	0	0	2	0.69	0	0	5	0.87
Total	288	100	288	100	288	100	288	100	576	100	576	100
Chi-Square			276.12				280.05				556.17	
*p*-Value			0.00 ***				0.00 ***				0.00 ***	

*** = *p* ≤ 0.001.

**Table 5:** Validity of OCI at Grade III (4°).

Answer	RIGHT				LEFT				ALL			
	Diagnosis	%	Direction	%	Diagnosis	%	Direction	%	Diagnosis	%	Direction	%
Correct	144	100	142	99	144	100	144	100	288	100	286	100
Error	0	0	2	1	0	0	0	0	0	0	2	1
Total	144	100	144	100	144	100	144	100	288	100	286	100
Chi-Square			136.11								280.056	
*p*-Value			0.00 ***								0.00 ***	

*** = *p* ≤ 0.001.

## Data Availability

The datasets generated or analyzed during this study are not publicly available due to being part of an ongoing thesis project. However, they are available from the corresponding author upon reasonable request.
